# Proceedings of the Virginia State Dental Association

**Published:** 1873-01

**Authors:** George F. Keesee

**Affiliations:** Secretary.


AMERICAN JOURNAL

OF

DENTAL SCIENCE.

Vol. VI. THIRD SERIES--JANUARY, 1873. No. 9.

### ARTICLE I.

*Proceedings of the Virginia State Dental Association.*

The third annual meeting of the Virginia State Dental Association was held in Richmond, at the dental rooms of Dr. Jud. B. Wood, on Wednesday, October 30th, 1872; the President, Dr. H. M. Grant, of Abingdon, in the chair. Quite a large number of dentists were present, representing every section of the State. After the usual preliminary business of reading the minutes of last stated and adjourned meetings, the President's annual address was postponed until Thursday evening. After a recess of five minutes, the Executive Committee reported favorably upon the following named gentlemen for membership, and they were unanimously elected: Drs. W. R. Dennis, Halifax C. H.; G. Smith, Louisa C. H.; R. M. Bidgood, Buckingham Co.; G. H. Hartman, Petersburg; C. F. Martin, Norfolk. A recess of five minutes was then taken to allow the newly elected members an opportunity to sign the constitution and pay the initiation fee. On the call for reports from the Standing Committees, Dr. Jas. F. Thompson, Chairman of the Executive Committee, said that he had nothing but a verbal report to present. The machinery of the Association had moved so harmoniously and quietly on, that he had nothing whatever to complain of; but on the other hand, had much to boast of—for the success of the Association had more than surpassed his expectations. Its roll of membership had been greatly augmented, and they were those who had taken hold with full purpose of entering, heart and soul, into the work ahead of them.

On motion of Dr. Thackston, the report was received and adopted.

The Committee on Dental Physiology reported, through its Chairman, Dr. Jas. Johnston, of Staunton, which report was received, and a vote of thanks tendered to Dr. Johnston for the paper as presented. The report entered into a minute detail of Physiology, with the enlightenments of the present day, and required the strictest attention of the members to strictly appreciate its true value and worth. Dr. Thackston regretted that comparatively little attention had been paid to Dental Physiology; many neglected it almost entirely; whereas, the dentist of to-day must thoroughly understand its details to fully measure up to the requirements of the profession and the public.

Dr. F. A. Jeter, of Richmond, Chairman of the Committee on Histology and Microscopy, stated that a lack of time and of favorable opportunities had prevented his preparing such a report as he wished to present, and in lieu thereof, by permission of the Association, read a very interesting paper from one of the Dental Journals, entering extensively into the microscopical characters of tartar, saliva, &c., and describing minutely the different species of animal life found therein.

Dr. Thackston continued the discussion by stating that the animalculæ were of different characters, depending upon the location where found—whether upon the enamel, dentine, cementum, or upon the mucous membrane of the mouth. Did not believe they acted on the tooth substance until the enamel had been softened by the acids of the mouth.

The Association then adjourned to meet at 8 o'clock, P. M.

EVENING SESSION.

At 8 o'clock the Association was called to order, the President in the chair.

The regular business was resumed. Dr. Henkel, Chairman of the Committee on Dental Therapeutics, stated that he had prepared no regular report, but read a report of a case which he had seen, in which a considerable portion of the alveolar process, extending from the lateral incisor to the second molar tooth, had been broken by undue force exerted in condensing a gold filling in the lateral incisor. Dr. Wayt thought the patient must surely have been mistaken in this having been the cause of her trouble, as he could hardly have supposed that lateral force sufficient to have produced such a fracture, could have been used in mere hand pressure in condensing a filling. But the patient insisted that she felt it break as the filling was being eondensed. On motion, the report was received.

Dr. Wood, Chairman of the Committee on Operative Dentistry, presented a lengthy report, accompanied with diagrams, both of which were highly instructive. The report spoke most favorably of Dr. Arthur's method of treating the teeth, setting forth its many advantages over the older method, and especially of enabling the operator to have a good view of fillings on approximal surfaces, so as ever afterwards to note the first recurrence of decay. The diagrams illustrated most favorably Dr. A's method, and also illustrated a case in which an inferior bicuspid which had been broken off by a blow to a line below the margin of the gum, had been restored to a condition as near its normal as could possibly have been supposed. The natural crown was made use of in the same way as Dr. Mack's artificial crowns are used. The report spoke at length upon the subject of capping nerves, and of the many substances used for that purpose, the most favorable being the oxychloride of zinc, or Guillois' cement. Dr. Johnston cautioned the Association against the too free use of oxychloride of zinc, or its synonyme, Guillois' cement, for capping of nerves. All would seem to work well for awhile, to the great satisfaction of both patient and operator; but if carefully examined at the expiration of twelve or eighteen months, the tooth would not be found in as healthy condition as previously supposed.

Without any complaint on the part of the patient, he had often, for his own gratification, (as he didn't mind hard work,) opened into some of the pulp cavities, and found that all was not working very harmoniously therein. Dr. Mahoney advised the introduction of Guillois' cement, with a pledget of cotton, and of packing it tightly in; whilst the report favored its introduction with the least possible pressure until after it began to harden, and then to be worked with a warm burnisher. Dr. Wayt believed it would not answer for permanent fillings, as they were anything else but permanent. Dr. Jeter had some cases on hand which he was watching with great care. Dr. Mills had been very successful in most cases, but had been sadly disappointed in one especial case. Dr. Thackston thought the results of capping of nerves with the articles under discussion might be summed up under three heads: First, That in which *just sufficient* irritation had been produced in the nerve to cause it to form secondary dentine. Secondly, That in which the nerve had been mummified, (for the want of a better term,) or completely dried. Thirdly, That in which the death and destruction of the nerve had been accomplished, together with all the symptoms favoring alveolar abscess. In its introduction he used it as dry as possible, as it was the liquid portion that possessed the escharotic properties.

The further discussion of the subject was postponed until the next evening. The President appointed Drs. Wood, Thackston and Moore to conduct the clinics for the following morning. The Association, after deciding to dispense with the next morning session, so as to allow the members to attend the State Fair, adjourned to meet the next evening at 7 o'clock.

### THURSDAY MORNING.

Pursuant to adjournment, quite a number of the members met at Dr. Wood's office at 9 o'clock. Dr. Wood performed three operations, which solicited flattering remarks from the older members present.

### EVENING SESSION.

The Association was called to order at 8 o'clock, the President in the chair. The discussion of the report on Operative Dentistry was resumed. The President, after taking the floor, spoke at length upon Alveolar Abscess. Had treated many cases by cleaning out the fang, and after gaining free access, injected carbolic acid and iodine; 5 parts of the former to 1 part of the latter. Had been less satisfactory than all other operations. Believed that in the majority of cases they were failures.

Dr. Mills used chromic acid with great success in the treatment of abscess, in the proportion of 10 grains of acid to 1 ounce of water. Dr. Johnston favored the use of the hyperdermic syringe. Often used glycerole of thymol; thought that one of the greatest sources of failure was the lack of patience on the part of the patient. Dr. Wayt thought that the general result was a failure. Dr. Moore said he had been very successful; had treated from 150 to 200 cases, and had only failed in one case, and that was one in which, in a previous operation, a piece of gold wire had been forced through the end of the fang, and which he could not remove. In this case he had to extract the tooth. His mode of treatment consisted in first cleansing the cavity well with warm salt water, removing all decay, cleaning out the fang, establishing a fistulous opening where one did not already exist, and treating thoroughly with carbolic acid. Did not believe there could well be too much medication. Had cured cases of ten years standing; had found the most favorable cases in the upper jaw. Always filled fang and crown at different sittings. The President, on again taking the floor, stated that the main difficulty seemed to exist in reaching the disease; but from the remarks made by Dr. Moore, felt much encouraged to persevere.

Dr. Moore used camphor (saturated solution) for bleaching teeth, applied on cotton. Dr. Thackston had abandoned the use of *all* anæsthetics on account of the great danger accompanying their use. Only used chloroform, to be applied locally in the socket, after extracting a tooth aching from alveolar abscess. Dr. Mills used carbolic acid for the same purpose. Dr. Moore had abandoned *entirely* the administration of chloroform as an anæsthetic. Dr. Grant used 40 or 50 grains of morphine to 1 ounce of chloroform, 1 ounce tincture of aconite, and 1 ounce alcohol for local application—to be used for the after pains of abscess. He used both chloroform and nitrous oxide in his practice. Dr. Johnston thought it criminal to administer chloroform; thought the Association should discountenance the administration of all anæsthetics.

The discussion of the report was then closed, and on motion of Dr. Keesee the reading of the President's annual address was then requested—the reading of which address most agreeably entertained the Association for a considerable length of time; and on motion of Dr. Wood, a unanimous vote of thanks was returned to Dr. Grant, the President.

Dr. Mills, Chairman of Committee on Dental Pathol[illegible] and Surgery, presented their report, which, on motio[illegible] received, filed and discussed.

The President appointed Drs. Wood, Scribner and Keesee a committee of three, to draft suitable resolutions expressive of the feelings of the Association upon the death of Dr. Wm. M. Dorsett, one of the founders of the Association.

Also a committee of five, consisting of Drs. Thackston, Moore, Johnston, Thompson and Grant, as a Committee on

Memorials, upon the death of Professor Thos. E. Bond, of the Baltimore Dental College.

On motion of Dr. Thackston, the annual contribution fee was made two dollars; ($2.00.) The Association then adjourned until 10 o'clock the next morning.

FRIDAY MORNING.

At 10 o'clock the Association was called to order, the President in the chair. The discussion of the report on Pathology and Surgery was continued. Dr. Thackston thought the report was highly instructive, and regretted that time did not allow a further discussion of at least its most important points.

Dr. Scribner, Chairmain of Committee on Mechanical Dentistry, presented a verbal report. Did not know of any improvements since the last session of the Association. vulcanite and celluloid were still used for cheap cases, and seemed to answer for the majority of cases. Dr. Johnston thought the celluloid was preferable to any other cheap base. His patients often preferred it to gold plate. Dr. Thackston had used the celluloid with much satisfaction; found it strong enough for partial cases. His patients thought it more comfortable than other bases; was adverse to having anything to do with the vulcanite. Dr. Jeter had used the whalebone rubber in preference to any other. Dr. Wood thought that air chambers were desirable. Dr. Grant stated that he had been experimenting with celluloid and was much pleased with it; had found it desirable for attaching the teeth to gold plates, and also for repairing vulcanite plates with. Drs. Mills and Johnston had both used the spiral springs for retaining vulcanite plates in flat mouths. Did not confine themselves to any particular mode of practice.

On motion, the discussion was closed.

Dr. Thompson, Chairman of Committee on Dental Literature, read a lengthy and able report, which was received and discussed.

Dr. Johnston thought that Dr. Lyons had issued a work which was well adapted for all ordinary purposes for the education of the public. Dr. Jeter thought that, in the absence of any standard pamphlet for the benefit of the public, all dentists should take especial pains in instructing parents as regards their children's teeth. Dr. Henkle thought it well to instruct them by frequent precepts—here a little and there a little. Dr. Keesee thought the Association should make use of the opportunity offered by the Dental Journals, and occasionally give to the profession the benefit of all cases of interest arising in their practice. Dr. Wood thought the Association should encourage the publication of a suitable pamphlet for the people. Dr. Thackston thought Dr. Keesee had struck the right mark, and thought that both young and old should make use of every opportunity to furnish articles for the chapter of "Hints and Queries" in the *Cosmos*, and the equally free pages of the "*American Journal of Dental Science.*"

On motion, the discussion was closed, and a vote of thanks returned to Dr. Thompson for the report.

Dr. Keesee, Chairman of the Committee on Voluntary Essays, reported that he had failed to receive by mail two essays which had been promised him, but instead thereof presented a report on "Transposition of the Teeth," occurring in the practice of Dr. Thompson, accompanied by a cast of the same, which was commented on for some length of time—the members stating what would be their mode of treatment for such a case.

The Association then proceeded to the election of officers for the ensuing year, which resulted in the election of

Dr. W. W. H. Thackston, of Farmville, as President. Dr. J. Hall Moore, of Richmond, First Vice President. Dr. James Johnston, of Staunton, Second Vice President. Dr. John W. Scribner, of Charlottesville, Third Vice President. Dr. Jud. B. Wood, of Richmond, Treasurer. Dr. George F. Keesee, of Richmond, Secretary.

On motion, the rules were dispensed with, and Drs. Grant, Thompson and Henkle were nominated for the Executive Committee, and they were unanimously elected·

Dr. Thackston was then duly installed as President of the Association for the ensuing year, and very gracefully thanked the Association for the honor conferred upon him.

On motion of Dr. Wood, a unanimous vote of thanks was offered to the retiring President, Dr. Grant, and to the Secretary, for the able manner in which they had performed the various duties assigned them during the past year. A unanimous vote of thanks was tendered Dr. Wood for the performance of his duties as Treasurer during the past, and for the use of his dental rooms for holding the present session. The Association then adjourned until 8 o'clock this evening.

EVENING SESSION.

The Association was called to order at 8 o'clock, the President in the chair. The President appointed the various Standing Committees for the ensuing year.

The Committee on Memorials presented their report, which was ordered to be spread upon the minutes, and copies of the same to be forwarded to the families of the deceased. In addition to which reports, the President paid a worthy and feeling tribute to the memory of the late Prof. Thomas E. Bond, of the Baltimore College of Dental Surgery.

On motion of Dr. Wood, a vote of thanks was offered to Dr. Robert Arthur, of Baltimore, for the publication of his book entitled "Treatment and Prevention of Decay of the Teeth," and its careful perusal recommended to all who have the interest and advancement of the profession at heart.

On motion of Dr. Thakcston, the Association heartily endorsed the action of the Southern Dental Association in the proposed erection of a monument, or some other enduring testimonial, to the memory of the late Dr. Chapin A.

Harris, and pledged to assume its fair and appropriate share of the labor and cost involved in the procurement and erection of said monument or other testimonial.

On motion of Dr. Wood, a vote of thanks was tendered to Samuel S. White, for his endeavors to defeat Josiah Bacon in the late suit of the Gardner Appeal Case.

Dr. John G. Wayt was elected as delegate to the Southern Dental Association. Drs. Johnston, Thackston, Scribner, Wood, Mahoney and Mills were elected as delegates to the American Dental Association. No further business appearing, the Association adjourned to meet in Richmond on the second Tuesday of December, 1873.

GEORGE F. KEESEE, *Secretary.*

